# Role of CT in the detection and management of cancer related complications: a study of 599 patients

**DOI:** 10.3332/ecancer.2023.1529

**Published:** 2023-04-05

**Authors:** Abhinav Bansal, Ekta Dhamija, Sheragaru Hanumanthappa Chandrashekhara, Ranjit Kumar Sahoo

**Affiliations:** Department of Radiodiagnosis and Medical Oncology, Dr BR Ambedkar Institute Rotary Cancer Hospital, All India Institute of Medical Sciences, New Delhi 110029, India

**Keywords:** cancer complications, oncoemergencies, CT, bleomycin toxicity, pulmonary damage, biliary obstruction, urinary tract obstruction

## Abstract

**Purpose:**

Cancer-related complications (CrC) and any potentially life-threatening findings detected on routine oncological imaging requires urgent intervention and needs proactive management. We conducted a retrospective study to highlight the role of imaging in the detection of CrC on computed tomography (CT)-scan while sharing our experience at a tertiary care cancer hospital.

**Materials and methods:**

All the reports of the CT scans performed in our department between January 2018 and December 2019 were reviewed and the imaging findings of CrC were recorded. Only the patients who had known malignancy and underwent imaging evaluation at our centre at baseline/follow up/surveillance were included. The clinical details of the patients were recorded and the findings were classified based on the system or organ involved and also on the basis of its impact on clinical management.

**Results:**

A total of 14,226 CT scans were performed during the study period, out of which 599 patients had CrC. Most of the CrC were seen involving thorax (265/599, 44.3%) followed by abdomen (229/599, 38.2%) and head and neck (104/599, 17.3%) regions. The commonly encountered CrC were pulmonary infections, superior vena cava obstruction and drug-induced lung changes.

**Conclusion:**

CrC have significant impact on the course of management of cancer patients and radiologist plays an important role in early diagnosis and initiation of prompt management of many such patients. CT is an excellent modality for early diagnosis of CrC which guides the oncologist for appropriate treatment.

## Introduction

Cancer has become one of the leading causes of morbidity and mortality in today’s world [1, 2]. Any acute potentially life-threatening event that occurs due to the malignancy or its treatment and requires urgent intervention can be labelled as an oncological emergency or complication. Such an event can occur at any time during the course of malignancy or sometimes can also be the initial presentation [3]. These can be attributed to direct effects of tumour progression or indirect effect. Direct effects include structural causes such as invasion or compression of adjacent viscera by the tumour. Indirect effects include metabolic and haematological changes which lead to systemic conditions like hypercalcaemia, tumour lysis syndrome, febrile neutropenia, hyperviscosity syndrome, and disseminated intravascular coagulation [4].

Imaging plays an important role in the detection of structural causes such as compression or infiltration of involved organs or haemorrhage or thrombosis of any major vessel [2]. Hence, the radiologist has to be vigilant to observe and inform about these findings to the treating physician in order to prevent their catastrophic effects [5]. Furthermore, incidentally detected pathologies like tumour rupture, ascites, pericardial effusion, pulmonary thromboembolism can have bearing on the treatment course in the form of modification or addition of drugs in the planned protocol. It is also imperative to differentiate and determine the treatment related changes like drug induced toxicity, which may have detrimental effect on patient outcome if not intervened at right time [6, 7].

Radiograph, ultrasonography (USG) and computed tomography (CT) are commonly used modalities used for diagnosis and evaluation of most of these conditions [1]. Magnetic resonance imaging (MRI), on the other hand, is primarily indicated for complications related to central nervous system (CNS) like metastasis, cerebral herniation, intracranial haemorrhage and spinal cord compression [2]. Ultrasound-, fluoroscopy- and CT-guided various interventional procedures are used for management of various structural complications of malignancy [8]; for instance, percutaneous trans hepatic biliary drainage (PTBD), percutaneous nephrostomy (PCN), percutaneous gastrostomy and stenting to relieve malignant luminal obstruction [9]. On the other hand, vascular interventions are carried out with curative or palliative intent in the settings of unresectable hepatocellular carcinoma, tumour rupture, tumoural haemorrhage, etc [2, 9]. This study has been conducted to highlight the role of CT in detection of cancer related complications (CrC) and oncological emergencies; while sharing our experience of tertiary care cancer hospital.

## Materials and methods

The retrospective study was conducted after obtaining approval from the institute ethics’ committee and the individual patient consent was waived off. The data had been collected from the CT reporting data base of our department.

All the reports of the CT scans performed between January 2018 and December 2019 were reviewed and the imaging findings of CrC were recorded. Only those patients, who had known malignancy and underwent imaging evaluation at our centre at baseline/follow up/surveillance were included. Out of total 14,226 CT scans performed on Somatom definition AS (Siemens, 64 slice scanner) during these 2 years, reports of 599 patient revealed imaging features of CrC and were included for further classification.

The demographic and clinical details of the patients were recorded and the findings were classified based on the system or organ involved and also on the basis of its impact on clinical management.

Classification of CrC based on the organ/system involved was broadly done under the groups of head and neck, chest and abdomen while the other classification was based on the impact of the finding under the categories as those warranting immediate intervention, elective intervention or which can be managed conservatively.

All the findings were then recorded and tabulated in word document. Continuous variables were presented as mean ± SD and categorical variables were presented as frequency and percentages.

## Results

A total of 14,226 CT scans were performed during the study period in our department, out of which 599 patients had CrC at the time of acquisition. Most of the CrC were seen involving the thorax (265/599, 44.3%) followed by abdomen (229/599, 38.2%) and head and neck (105/599, 17.3%) regions (Table 1).

**Figure 1.**
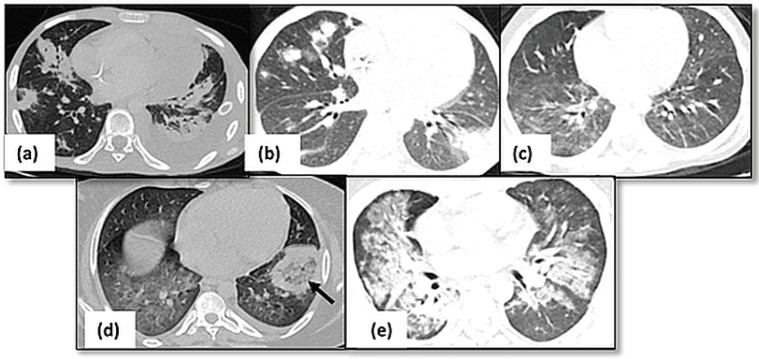
Pulmonary infections. (a): Axial HRCT image showing multiple consolidations in bilateral lungs with left sided pleural effusion suggestive of bacterial pneumonia. (b): Fungal infection with multiple nodules having surrounding ground glass opacity in bilateral lungs. (c): Axial HRCT image of a patient with viral pneumonia showing patchy areas of ground glass opacity in bilateral lungs. (d): Axial HRCT image showing presence of consolidation with central ground glass opacity (arrow) in left lower lobe suggestive of mucormycosis. (e): Consolidation and ground glass opacity seen in perihilar and peribronchovascular locations s/o PCP pneumonia.

**Figure 2.**
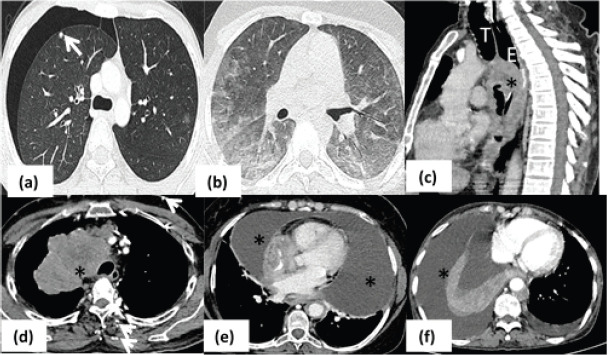
Pulmonary complications. (a): Axial HRCT image in a patient with osteosarcoma showing presence of subpleural metastatic nodule (arrow) in right upper lobe with right sided pneumothorax. (b): Axial HRCT image showing peripheral and lower lobe predominant ground glass opacities in bilateral lung fields in a case of Hodgkin’s lymphoma on chemotherapy s/o bleomycin toxicity. (c): Sagittal CT image of patient with esophageal cancer (asterisk) demonstrating airway invasion anteriorly (arrow). (d): Conglomerate mediastinal mass (asterisk) proven as lymphoma is seen to cause complete obliteration of SVC with multiple chest wall collaterals (arrows) – SVC syndrome. (e): Axial mediastinal window of a patient with leukemia who had massive pericardial effusion (asterisk) causing respiratory distress. (f): Axial CT iamge of lung cancer patient with gross right sided pleural effusion (asterisk) causing collpae of underlying lung. (T- trachea; E- esophagus).

Thoracic complications: CT thorax identified the CrCs predominantly as pulmonary infections (62/265, 23.4%), superior vena cava (SVC) obstruction (40/265, 15.1%) and drug induced lung changes (34/265, 12.8%). Pulmonary complications in the form of infections (62/265, Figure 1), haemorrhage (14/265) and All-trans retinoic acid (ATRA) toxicity (3/265) were seen in patients with leukaemia while lymphoma patients had Bleomycin induced CrC (16/265). Bacterial infections were seen as airspace nodules or consolidation, whereas angioinvasive fungal infections seen as ground glass opacification. The primary lung cancer (*n* = 15) and mediastinal masses (*n* = 19) including thymic malignancies and lymphoma, were the common causes of vascular infiltration and SVC syndrome (Figure 2).

**Figure 3.**
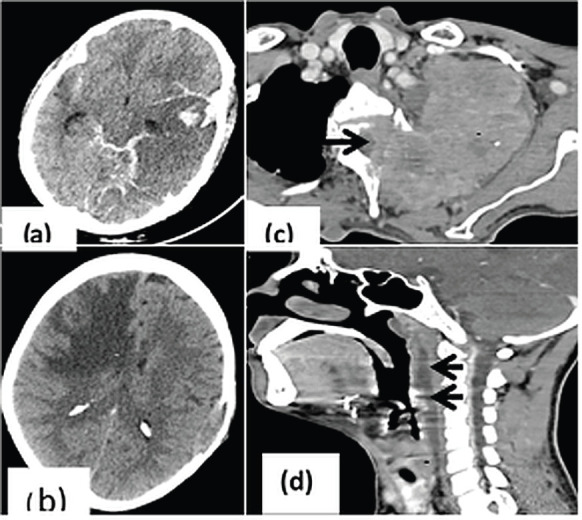
CrC in head and neck region. (a): CT head showing parenchymal as well as subarachnoid bleed in a patient with acute myeloid leukemia; (b): Unenhanced CT showing significant white matter edema (asterisk) in right fronto-parietal region causing mass effect on ipsilateral frontal horn of lateral ventricle; (c): Axial CT image showing pancoast tumour (asterisk) in upper lobe of left lung causing cervical vertebral erosion and intraspinal extension (arrow); (d): Post radiation edema in patient with glottic carcinoma (asterisk) with fluid collection in the retropharyngeal space (arrows) indicating immediate intervention for the same before commencing further treatment.

CNS involvement (Figure 3): Intra-parenchymal brain metastases with oedema (40/105, 38.1%) were the common cause for patient’s presentation to hospital with CNS symptoms like headache and seizures. Brain metastases were majorly seen in patients with carcinoma lung (*n* = 24) and breast (*n* = 5). Significant brain herniation needing immediate neurological intervention was seen in metastases (*n* = 6) with oedema, intracranial haemorrhage (*n* = 4) or infarct (*n* = 3). Perineural spread of disease and cavernous sinus involvement was seen in 14/105 (13.3%) patients, majorly with head and neck tumours.

**Figure 4.**
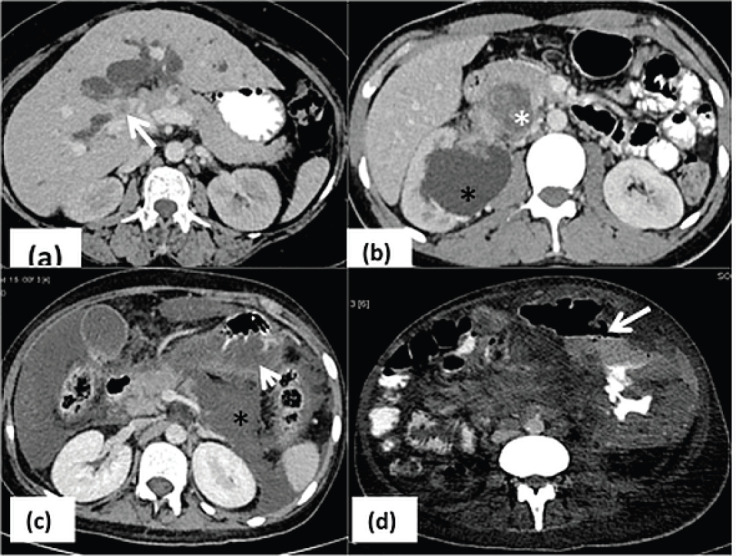
Abdominal complications. (a): Hyperbilirubinemia due to hilar mass (arrow) causing significant dilatation of intrahepatic biliary radicals which delays initiation of chemotherapy until relieved with percutaneous drainage; (b): right sided hydronephrosis (black asterisk) due to obstruction by the retroperitoneal mass (white asterisk); (c): L–asparaginase induced pancreatitis seen as necrosis in the pancreatic parenchyma with fluid collection (asterisk) and edema in the adjacent stomach wall (arrow); (d): axial CECT image in a patient with lymphoma with bowel thickening and aneurysmal dilatation of jejunal loop with focal defect in bowel wall (arrow) suggestive of perforation.

Abdominal complications (Figure 4): Malignant urinary tract obstruction (57/229, 25%) was caused predominantly by carcinoma cervix (*n* = 13), germ cell tumours (*n* = 12), carcinoma ovary (*n* = 7) and carcinoma bladder (*n* = 9). Biliary obstruction was seen in 29/229 (12.6%) patients of cholangiocarcinoma, periampullary carcinoma and carcinoma gall bladder, out of which four patients developed cholangitis requiring urgent PTBD. Intestinal obstruction was seen in 34/229 (14.8%) patients-due to post op adhesions or inflammation (*n* = 17), post Radiotherapy (RT) changes (*n* = 7) and obstruction due to mass or serosal metastases (*n* = 10). The causes of bowel perforation include tumour infiltration (*n* = 5), post inflammatory and ischemic bowel perforation (*n* = 4). Enterocolitis included both radiation induced (*n* = 12) and infective enterocolitis (*n* = 15). Pseudoaneurysms (*n* = 3), tumour bleed (*n* = 4), ruptured hepatic metastasis (*n* = 1) led to intra-abdominal haemorrhage warranting immediate intervention (Table 2).

Complications in various malignancies: CNS complications were most commonly encountered due to perilesional oedema in brain metastasis in patients with lung (24/40) and breast (5/40) cancer. Pulmonary infections were a common cause of hospital presentation in patients with leukaemia (46/62). Patients with lymphoma had drug-induced lung injury (16/31) and SVC syndrome (12/40). Formation of bronchoesophageal fistula (*n* = 6) and aspiration (9/19) were most common cause of morbidity in patients with carcinoma oesophagus. Pelvic fistula formation (*n* = 16) and bowel obstruction (*n* = 17) were the most common CrC in patients with colorectal carcinoma.

Treatment related changes: Drug induced changes (*n* = 42) include pulmonary complications (34/42), chemotherapy induced pancreatitis (5/42) and pseudocirrhosis (3/42). Among the post RT complications (*n* = 34), RT induced enteritis (12/34) and bowel obstruction (7/34) were most common. Post-operative adhesions leading to bowel obstruction was the most common surgery related complication (17/24).

**Figure 5.**
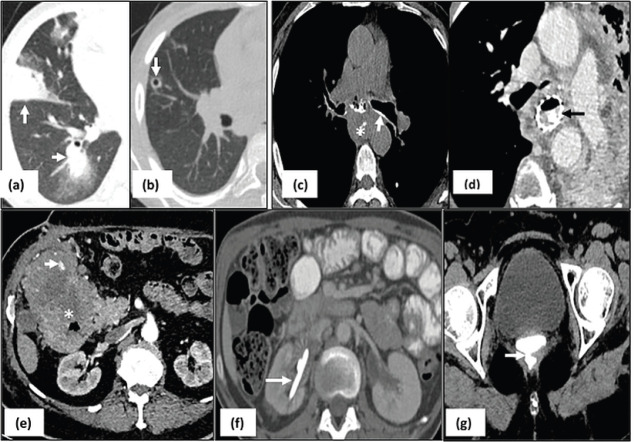
Interventions for management of CrC:leukemia patient with fungal pneumonia on HRCT image (arrows in a) responded to antifungal drugs evident as resolution of nodules and ground glass opacities with appearance of internal cavitation (arrow in b). Another patient having carcinoma esophagus with formation of trachea-esophageal fistula seen as oral contrast tracking in the airways (arrow in c) underwent esophageal stenting (arrow in d). Similarly, patient with intratumoural bleed (arrow) had to undergo embolisation for hemodynamic stability (e) and PCN (arrow in f) was done for urinary diversion in patient with hydronephrosis (same patient as in Figure 1b) before chemotherapy in order to improve the renal function status. On the other hand, post radiation rectovaginal fistula in a female with carcinoma cervix (arrow in g) was kept under close surveillance with conservative approach.

CrC classification based on need of intervention: There were 355 (59.4%) CrC that needed either immediate medical, surgical or radiological intervention, which included ICT placement for pneumothorax; laparoscopy or open surgery for intestinal obstruction; treatment of pulmonary infection, which also led to interruption of routine treatment; etc. as documented in Table 3. Where chemotherapy or radiation induced changes (*n* = 124) required change in management strategy; complications like pulmonary haemorrhage (*n* = 14) or pulmonary thromboembolism (*n* = 13) needed additional treatment before proceeding with routine regime. The conditions like asymptomatic intussusceptions (*n* = 4) and radiation induced or tumour induced pelvic (vesicovaginal or rectovaginal, Figure 5g) fistulas (*n* = 6) were managed conservatively.

## Discussion

Any potentially life-threatening event in cancer patients that occurs due to the malignancy or its treatment is considered an oncological emergency which needs early and aggressive management. Thus, it becomes imperative for the radiologist to identify and promptly communicate to the treating clinician for early intervention. They can be broadly classified as metabolic, haematological and structural conditions [10]. Metabolic and haematological emergencies including hypercalcaemia, hyponatremia, hyper viscosity syndromeor disseminated intravascular coagulation are mainly diagnosed in relation to laboratory findings [11]. Imaging especially cross-sectional imaging is performed to rule out structural causes of the symptoms. Multidetector CT is the most readily performed imaging modality for cancer patients at baseline, during treatment and post treatment follow up. In addition to the information related to the status of primary tumour, it is able to identify the sequel or changes due to the tumour or metastases or treatment being administered for the particular cancer.

In this study, we were able to highlight that, thoracic complications (265/599, 44.3%) were the most common CrCs which might be due to early presentation with debilitating symptoms like dyspnoea, fever, cough, hemoptysis which hampers patient’s day to day life. Not only does the CT helps by ruling out underlying primary lung carcinoma but is also able to illustrate other infective or non-infective causes of the symptoms. Pulmonary infection (62/265, 23.4%) was the most common thoracic complications seen in our patients predominantly in patients receiving treatment for lymphoma and leukaemia (Figure 1). Presence of active infection, especially in post–marrow transplant patients, indicates addition of the antibiotics, antifungals or antivirals with interim discontinuation of the routine therapy (Figure 5a and b). On the other hand, pulmonary haemorrhage would need treatment with steroids which would need continuous monitoring of the patient.

The other causes of dyspnea include pleural or pericardial effusion which would warrant immediate therapeutic drainage if compromising quality of life of the patient. Similarly, the chemotherapeutic agents like Bleomycin induce acute interstitial pneumonia which the oncologist should be aware of before planning the next regime. On the other hand, differentiation syndrome due to ATRA can present as features of pulmonary oedema leading to discontinuation of the drug [6]. Pneumothorax due to subpleural cavitatory metastases can lead to sudden deterioration of patient’s clinical status and need immediate drainage with intrathoracic chest tube placement [12]. On the other hand, structural causes like SVC syndrome would need immediate initiation of chemotherapy to reduce symptoms and at times, patient might need balloon dilatation of the vein (Figure 2).

Image guided interventions for cancer patients can be performed for treatment or palliative therapy. Obstruction of biliary system leads to hyperbilirubinemia which needs to be drained with PTBD so as to make patient fit for chemotherapy and cancer control. MDCT can identify the level and sequel of malignant obstruction of biliary, urinary, bowel and vascular system [13]. Urinary tract obstruction may progress to uremia and would need immediate diversion with PCN tube or a ureteric stent using USG and fluoroscopy guidance (Figure 5f). However systemic treatment post-stenting, cystoscopic evidence of bladder invasion and length of obstruction greater than 3 cm are associated with stent failure making PCN placement the preferred option in these conditions for decompression [14].

Similarly, the neurological symptoms caused due to brain oedema, cerebral herniation or cord compression are better delineated with MRI owing to its superior resolution; however, in our resource constraint setting, it is not possible to perform MRI in every patient and CT is preferred to rule out secondary changes of herniation and hydrocephalus [15]. Radiation induced laryngeal oedema can cause severe airway in 15%–50% patients warranting immediate intervention [16].

Abnormal fistulous communication between the oesophagus and the trachea, bronchi, and lung parenchyma occurs in lymphoma, oesophageal, and lung malignancies causing recurrent pulmonary infections and aspiration pneumonitis [17]. Site and number of fistulae as well as condition of underlying lung can be easily assessed with the help CT and new reconstruction algorithms like virtual endoscopy. This would need measures for rerouting the food intake of the patient by means of Ryle’s tube, percutaneous gastrostomy or jejunal gastrostomy. Some patient would benefit from oesophageal and tracheal stent (Figure 5c and d); thus it becomes important for radiologist to highlight such complications in the report; in addition to staging of the carcinoma; so as to improve the quality of life of the patient.

Rupture of hypervascular malignancies such as hepatocellular carcinoma, renal cell carcinoma and melanoma or splenic rupture in cases of hematological malignancies are well known causes of hemoperitoneum. The interpreting radiologist can highlight the cause of active contrast extravasation which can help in planning embolisation and achieving haemostasis (Figure 5e) [18]. Hypercoagulable state in malignancy due to prothrombotic factors released by cancer cells has been implied in thrombosis and infarct related acute emergencies such as mesenteric ischemia and solid organ infarct [19]. Multiphase CT can demonstrate the primary mass, status of mesenteric vessels, and any associated abnormal enhancement or bowel thickening [4]. Chemotherapy induced pancreatitis is most commonly associated with L-asparaginase (Figure 4c) used for treatment of leukaemia and sorafenib used for metastatic RCC [6]. Patient usually presents with abdominal pain within 3–4 weeks of initiation of therapy and it becomes imperative to obtain clinico-radiological correlation in order to prevent further complications associated with pancreatitis. Leakage commonly occurring at anastomotic site, is seen in approximately 4% of patients following urinary diversion, and was seen in 4 of our cases [20]. An evaluation of excretory phase images is essential in patients with increased output from a drainage catheter or urinary drainage from the wound that might need re-exploration

Our study concurs with previous studies highlighting the important role of CT in detection of CrC [10]. In addition, it could illustrate the impact and additional role of CT in cancer patients which extends beyond staging and response assessment. It emphasises the role of radiologists in providing paramount information to the treating physician which has crucial effect on the patient management. The novelty of this study lies in categorisation of the various conditions according to the need and type of intervention required in that particular scenario, with appropriate clinico-radiological correlation; which the reporting physician should be cognizant about. However, it is limited by its retrospective study design conducted in a single centre. It is a descriptive study highlighting the findings seen only on one imaging modality. The impact of management taken into consideration was based on overall general approach whereas actual intervention would differ from patient to patient based on the multidisciplinary team meetings.

## Conclusion

To conclude, the study highlights the huge burden of CrC in cancer patients and thus, emphasises the role of imaging in patient assessment. CrC needs early detection and treatment and a radiologist must be familiar with the imaging features and implications so as to timely inform and coordinate with the treating physician. The radiologist must have adequate knowledge of complications associated with cancer including post treatment complications, so that subtle complications are not missed and promptly communicated to the disease management group which can have a grave impact on the further treatment regime of the patient.

## Author contributions

Abhinav Bansal- Manuscript preparation, Data collection and analysis

Ekta Dhamija- Concept, Design, Data analysis, Manuscript editing

Chandrashekhara SH- Manuscript editing and review

Ranjit Kumar Sahoo- Concept, Manuscript editing and review.

## Conflict of interest

Nil.

## Funding

None.

## Ethical approval

Institute ethics committee approval for this study was obtained vide letter number IEC-1194/04.12.2020, RP-01/2021.

## Figures and Tables

**Table 1.** CrC detected in CT head, neck and chest.

System	Complication	Number (*n*)
Head and neck	Brain metastases with significant perilesional oedema	40
	Cavernous sinus involvement	14
	Haemorrhage	11
	Perineural spread	8
	Airway compromise	8
	Osteoradionecrosis	8
	Cellulitis and retropharyngeal abscess	7
	Post RT laryngeal oedema	5
	Infarct	3
	RT induced sarcoma	1
Total		105
Thorax	Infection	62
	SVC obstruction	40
	Drug induced changes	31
	Spinal cord compression	24
	Aspiration	19
	Pericardial/pleural effusion	24
	Pulmonary haemorrhage	14
	Pulmonary embolism	13
	Pneumothorax	13
	Pulmonary oedema	13
	Bronchoesophageal fistula	6
	ATRA differentiation	3
	GVHD	2
	Fat embolism	1
Total		265

RT: Radiotherapy; SVC: Superior vena cava; ATRA: All-trans retinoic acid; GVHD: Graft versus host disease

**Table 2.** CrC detected on abdominal CT scans.

System	Complication	Number (*n*)
Abdomen	Ureter obstruction	57
	Intestinal obstruction	34
	Fistula formation	22
	Enterocolitis	27
	Biliary obstruction	29
	Hepatic/renal/splenic infarct	11
	Bowel perforation	9
	Pseudoaneurysm/tumour bleed	8
	Panniculitis	7
	Post op urine leak	4
	Chemotherapy induced pancreatitis	5
	Intussusception	4
	Pseudocirrhosis	3
	EHPVO	4
	SMA syndrome	2
	Hepatosplenic candidiasis	2
	Bowel anastomotic leak	1
Total		229

EHPVO: Extrahepatic portal venous occlusion; SMA: Superior mesenteric artery

**Table 3.** CrC based on the interventions needed.

Intervention	Complications	Number (*n*)
Needed immediate surgical or medical intervention	Pulmonary infection SVC obstruction Intestinal obstruction Neutropenic enterocolitis Spinal cord compression Airway compromise/obstruction Brain herniation Pneumothorax Anastomotic site leak Cellulitis or retropharyngeal Abscess/ oedema GVHD Engraftment syndrome	62 40 34 27 24 8 13 13 7 6 2 1
Radiological intervention	Ureteric obstruction Biliary obstruction Gross effusion Tumour rupture/bleed Intratumoural bleed	57 29 24 7 1
Changed the treatment strategy	Drug induced complications Treatment related changes (post RT, post-surgery complications) Spinal cord compression Perineural spread of disease	42 58 24 8
Required additional treatment course	Pulmonary infection Pulmonary haemorrhage Pneumothorax Pulmonary thromboembolism Tumour bleed Haematuria post RT which did not respond to conservative management	62 14 13 13 8 1
Wait and watch	Fistula formation Visceral infarct Pseudocirrhosis (Transient) Intussusception	22 11 3 4

SVC: Superior vena cava; GVHD: Graft versus Host Disease
